# Serum creatine kinase levels are not associated with an increased need for continuous renal replacement therapy in patients with acute kidney injury following rhabdomyolysis

**DOI:** 10.1080/0886022X.2022.2079523

**Published:** 2022-05-24

**Authors:** Liuniu Xiao, Xiao Ran, Yanxia Zhong, Yue Le, Shusheng Li

**Affiliations:** a Tongji Medical College of Huazhonng University of Science and Technology, Wuhan, China; b Department of Intensive Care Unit of Tongji Hospital Affiliated to Tongji Medical College of Huazhong University of Science and Technology, Wuhan, China

**Keywords:** Rhabdomyolysis, acute kidney injure, continuous renal replacement therapy, creatine kinase

## Abstract

Severe rhabdomyolysis can lead to acute kidney injury (AKI). Previous studies have reported a benefit from continuous renal replacement therapy (CRRT) for rhabdomyolysis-associated AKI. Here, we investigated the potential for serum creatine kinase (CK) levels to be used as a marker for CRRT termination in patients with AKI following rhabdomyolysis. We compared different CK levels in patients after CRRT termination and observed their clinical outcomes. We retrospectively collected 86 cases with confirmed rhabdomyolysis-associated AKI, who were receiving CRRT in Tongji Hospital. Patients’ renal functions were assessed within 24 h of intermission, patients with urine output ≥ 1,000 mL and serum creatinine ≤ 265 umol/L were considered for CRRT termination. After termination, 33 patients with a CK > 5,000 U/L were included in an experimental group, and 53 patients with a CK < 5,000 U/L were included in a control group. Clinical outcomes were compared between the two groups. Higher CK levels, as well as worse renal functions, predicted the necessity of CRRT. After CRRT termination, the in-hospital mortality (*p* = 0.389) and Multiple Organ Dysfunction Syndrome (MODS) incidence (*p* = 0.064) were similar between the two groups, while the experimental group showed a significantly shorter in-hospital length of stay (*p* = 0.026) and Intensive Care Unit (ICU) length of stay (*p* = 0.038). CRRT termination may be independent of CK levels for patients with rhabdomyolysis-associated AKI, and this is contingent on their renal functions having recovered to an appropriate level.

## Introduction

Rhabdomyolysis (RM) describes a syndrome caused by the disintegration of skeletal muscle tissues, leading to leakage of various intercellular myocyte contents into the bloodstream [1]. The etiology of muscle injury in rhabdomyolysis is diverse, including direct trauma, toxins, infections, and strenuous exercise [2]. The clinical symptoms of RM vary significantly, from an asymptomatic increase in serum levels of enzymes released from damaged skeletal muscles to obvious muscle weakness, myalgia, and dark urine [3]. Severe cases are characterized by the metabolic syndromes, including volume depletion, electrolyte abnormalities and even acute kidney injury (AKI).

A diagnosis of RM is given when there are severe muscle symptoms with creatine kinase (CK) levels >1,000 U/L or greater than five times the normal limit [4]. Serum levels of CK correlate with the severity of RM [5]: Moderate RM is defined as CK levels within 5,000–15,000 U/L, and severe cases are above 15,000 U/L. A recently published review concluded that increasing levels of CK, or failure of levels to decline despite therapies, suggest an ongoing muscle injury and development of AKI [3].

AKI is a serious and common complication of RM, occurring in 13%–50% of patients [6], and up to 7%–10% of AKI is attributed to RM [7]. In critically ill patients, AKI doubles mortality with a rate of 59%, compared to 22% in patients without AKI [8]. Although the pathogenesis of RM–induced AKI is not fully elucidated, previous experimental evidence suggests that renal vasoconstriction, direct and ischemic tubule injury, and tubular obstruction are three major mechanisms involved in disease progression [9].

The myoglobin released from damaged muscles plays a dominant role in the pathogenesis ofRM–induced AKI [10]. Myoglobin is an iron-containing small protein with a molecular weight of 17.8 kDa and exists at a low concentration in serum under physiological conditions [11]. However, for a skeletal muscle injury, serum myoglobin levels increase within one hour and return to a normal range within one to six hours after lesion resolution. Myoglobin is freely filtered by the glomerulus and absorbed in the proximal tubule by endocytosis [12]. Excess myoglobin released into the circulation will lead to myoglobinuria and renal insufficiency, which brings about renal tubular obstruction and oxidative injury. A persistently increased serum creatinine, as well as a decreased glomerular filtration rate (eGFR), predicts deterioration of renal functions, resulting in AKI.

Since the molecular weight of myoglobin is relatively large and difficult to remove by conventional low permeable membranes, myoglobin could be eliminated most effectively by a continuous renal replacement therapy (CRRT), as compared with traditional intermittent dialysis [13]. Previous studies have reported significant benefits for patients with RM-associated AKI who received early CRRT, including reduced incidence of MODS, lower levels of inflammatory factors, and rapid recovery of renal function [14–17].

For patients with AKI, the termination of CRRT often depends on the patient’s renal function at intermission. For example, once urine output is above 1,000 mL, and serum creatinine is below 265 μmol/L within 24 h at intermission, CRRT could be considered for suspension [18]. However, in light of RM-associated AKI, the higher level of CK predicts an ongoing muscle injury, as well as a potentially increased risk of renal dysfunction. Whether the CK levels should be considered for CRRT termination is controversial. Therefore, we performed a retrospective study to compare different CK levels at CRRT termination and to identify its impact on clinical prognosis.

## Materials and methods

### Data collection

For a retrospective study, we collected 86 cases with confirmed RM-associated AKI, who were received CRRT from January 1st of 2012 to December 31st of 2020 in Tongji Hospital Affiliated with Tongji Medical College of Huazhong University of Science and Technology. The diagnostic criteria for RM were serum CK levels of >1,000 U/L, along with clinical symptoms, such as muscle weakness, myalgia, or dark-colored urine. All patients underwent urinalysis (dipstick and microscopy) to differentiate between hematuria and myoglobinuria, and positive urine dipstick tests were confirmed by the absence of red blood cells in the urine sediment.

AKI was defined using a modified Risk, Injury, Failure, Loss, End Stage Renal Disease (RIFLE) classification system, based on eGFR criteria. All patients were stratified as “Risk” if the serum creatinine value was 1.5X greater than baseline, “Injury” if the value was 2X times greater, and “Failure” if the value was 3X greater [4]. Traditional treatments of RM-associated AKI focused on classic protective measurements, including early and aggressive fluid resuscitation, alkalinization of urine, and forced diuresis by osmotic agents. CRRT was applied to patients with at least one of the following criteria: (1) oliguria (urine output < 400 mL/D) or anuria (urine output < 100 mL/D), for more than two days, (2) symptoms of uremia, such as vomiting, apathy, irritability or drowsiness, (3) blood PH <7.15, or serum bicarbonate ion <15 mmol/L, (4) blood urea nitrogen >17.8 mmol/L, or serum creatinine ≥442 μmol/L, (5) obvious electrolyte disturbances, such as serum potassium ≥6.5 mmol/L or sodium ≥160 mmol/L.

### Standardized treatments with CRRT

All participants underwent early and vigorous fluid resuscitation to the prevention of shock. Sodium bicarbonate therapy was replenished for patients with obvious metabolic acidosis (blood PH < 7.15). CRRT was either continuous venovenous hemodialysis (CVVHD) or continuous venovenous hemodiafiltration (CVVHDF). CVVHD used standard capillary hemofilter (AV 600S; Fresenius Medical Care, Bad Homburg, Germany; blood flow of 150 mL/min, dialysate exchange rate 1,000 mL/min). CVVHDF also used the above hemofilter (AV 600S; Fresenius Medical Care, Bad Homburg, Germany; blood flow of 180 mL/min, dialysate exchange rate 1,500 mL/min). The average CRRT period was 16 h, and intermission was 24 h. Ultrafiltration was adjusted according to clinical need. All materials were employed within the limits of intended use.

### Study population

For a retrospective analysis, the demographic characteristics, etiology, Acute Physiology and Chronic Health Evaluation II score (APACHEII), McMahon score, and laboratory examinations for 86 cases with RM-associated AKI were collected from electronic medical records. According to the British Renal Association guidelines for AKI, we chose urine output and serum creatinine at intermission, as indicators for monitoring renal function recovery. Once the urine output reached >1,000 mL, and a serum creatinine ≤265 μmol/L, within 24 h of intermission, CRRT was terminated.

For patients with a confirmed RM-associated AKI, serum CK levels were dynamically monitored through the full course of CRRT therapy, since the CK levels could reflect the disease process, as well as therapeutic effects. Patients with different serum CK levels after CRRT termination were included into two groups: The experimental group (33 patients) (CK levels still >5,000 U/L after termination), and the control group (53 patients) (CK levels <5,000 U/L after termination). In the history of RM classification, levels of CK have been thought of as a key factor in predicting the risk of kidney injury. Higher CK levels after termination might predict a non-overt sustained RM, despite standard CRRT treatment. Patients with moderate or severe RM (CK levels >5,000 U/L) suffer from a potential development of renal failure, which could potentially lead to a need for CRRT again. Different clinical prognoses were compared between the two groups to explore whether the level of CK should be considered as an indicator for CRRT termination. For patients with severe RM after CRRT and CK levels above 15,000 U/L, a subgroup analysis was conducted to discuss the relationship of these factors with outcomes, since these participants still showed a potential increased risk of dialysis by standard RM classification.

### Outcome measurements

The primary clinical outcome of this research was in-hospital mortality, while secondary outcomes included in-hospital length of stay, ICU length of stay, length of CRRT, and MODS incidence. Laboratory examinations, including routine blood tests, CK, myoglobin, renal function variables, and inflammatory indicators, were conducted for RM-associated AKI on admission, as well as at the time of CRRT termination. Correlations between CK and other variables were analyzed after termination.

### Statistical analysis

Continuous variables were presented as mean ± standard deviation and checked by Student’s *T*-test. Categorical variables were displayed as frequencies and percentages and analyzed by Chi-square (two-sided). Linear correlations between different variables were determined by Spearman rank correlation analysis. *p* < 0.05 was set as the cutoff for statistical significance, and all data management and analyses were performed using Graphpad Prism 6.0.

## Results

### Baseline characteristics of patients with RM associated AKI

From January 1st, 2012 to December 31st, 2020, 86 patients with RM- associated AKI underwent CRRT in our hospital. The demographic characteristics of study participants are presented in Table 1. The average age was 43 years, and approximately half were male. Reasons for RM included: infections (36.2%), toxins (26.7%), strenuous exercise (15.1%), heat stroke (15.1%), and trauma (6.9%). The mean McMahon score was seven, which predicted a sensitivity and specificity risk for CRRT. The APACHEII score for all subjects was within 16–23. After CRRT termination, 33 patients with CK levels above 5,000 U/L were included to an experimental group, and the remaining 53 cases were included to a control group.

**Table 1.** Demographic characteristics of patients with RB-associated AKI.

Patient characteristics	*N* % or median [Range: Min Max]
Age, mean, y	43 [18, 86]
Sex, male, *n* (%)	55 (63.9)
Etiology, *n* (%)	
Trauma	6 (6.9)
Toxins	23 (26.7)
Infections	31 (36.2)
Strenuous exercise	13 (15.1)
Heat stroke	13 (15.1)
McMahon score	7 [5, 10]
APACHEII score	19 [16, 23]

APACHEII Score: Acute Physiology and Chronic Health Evaluation II Score.

### Laboratory markers on admission

Baseline laboratory markers for 86 cases were collected on admission (Table 2). First, there were no differences observed between the APACHEII and McMahon scores. Secondly, the results of this study showed that leukocytes, neutrophils, lymphocytes, and hemoglobin were comparable between the two groups (all *p*-value > 0.05). In addition, CK and myoglobin, which represented a condition of muscle injury, were also similar (*p* = 0.081, *p* = 0.103). Lastly, the average urine output was below 400 mL/d, and the serum creatinine was 497.2 μmol/L. These two groups displayed a similar state of renal function and belonged to the “Failure” stage according to RIFLE classification (Urine output: *p* = 0.098, Creatinine: *p* = 0.344, Urea Nitrogen: *p* = 0.072, eGFR: *p* = 0.183). There was no difference observed for inflammatory biomarkers, Procalcitonin (PCT) and C-Reaction Protein (CRP), between the two groups (*p* = 0.863, *p* = 0.859).

**Table 2.** Laboratory examination of patients with RM-associated AKI on admission.

Variables	All (*n* = 86)	C*K* > 5000 U/L (*n* = 33)	C*K* < 5000 U/L (*n* = 53)	*P* value
APACHEII score	18.92 ± 0.668	18.72 ± 0.535	19.11 ± 0.801	0.633
McMahon score	7.26 ± 0.418	6.14 ± 0.199	7.37 ± 0.636	0.831
Leukocyte (× 10^9^/L)	14.38 ± 1.195	15.08 ± 1.233	13.94 ± 1.157	0.520
Neutrophils (× 10^9^/L)	8.516 ± 1.191	8.430 ± 1.523	8.651 ± 0.858	0.177
Lymphocyte (× 10^9^/L)	1.101 ± 0.090	1.173 ± 0.094	1.029 ± 0.085	0.367
Hemoglobin (g/L)	114.9 ± 5.494	120.4 ± 6.650	111.4 ± 4.338	0.239
CK (U/L)	11088 ± 1494	13419 ± 1546	9566 ± 1442	0.081
Myoglobin (ng/mL)	1169 ± 32.87	1213 ± 39.84	1138 ± 25.89	0.103
Urine output (mL/d)	368.6 ± 46.13	395.2 ± 53.87	327.2 ± 38.39	0.098
Creatinine (umol/L)	497.2 ± 29.54	474.6 ± 26.65	515.8 ± 32.43	0.344
Urea nitrogen (mmol/L)	16.89 ± 1.935	13.76 ± 2.096	18.85 ± 1.775	0.072
eGFR (mL/min/L)	46.27 ± 7.158	55.01 ± 8.114	41.26 ± 6.201	0.183
PCT (ng/mL)	2.368 ± 0.492	2.296 ± 0.561	2.416 ± 0.423	0.863
CRP (mg/L)	62.43 ± 12.69	64.31 ± 12.53	61.03 ± 12.85	0.859

CK: Creatine Kinase; eGFR: Glomerular Filtration Rate; PCT: Procalcitonin; CRP: C-Reaction Protein.

### Variables after CRRT termination

After CRRT termination, patients in the two groups showed a similar APACHEII score, as well as a McMahon score (*p* = 0.742, *p* = 0.355). The mean McMahon score was four points for all participants, which predicted a lower potential risk of CRRT. Importantly, the level of myoglobin was observed to be significantly higher in the experimental group (852.1 ± 79.14 vs 579.7 ± 63.66, *p* = 0.010), in addition to markedly higher CK levels. Notably, urine output, serum creatinine, urea nitrogen, and eGFR, which reflected the recovery of renal function, were comparable (*p*-value > 0.05). All patients reached the goal of urine output of > 1,000 mL/D, with serum creatinine ≤ 265 μmol/L after CRRT. In addition, the inflammatory response was similar, and under a controllable condition (*p*-value > 0.05) Table 3.

**Table 3.** Variables of patients with RM-associated AKI after CRRT termination.

Variables	All (*n* = 86)	C*K* > 5000 U/L (*n* = 33)	C*K* < 5000 U/L (*n* = 53)	*P* value
APACHEII score	16.55 ± 0.305	16.34 ± 0.338	16.72 ± 0.272	0.742
McMahon score	4.68 ± 0.221	5.17 ± 0.242	4.22 ± 0.199	0.355
Leukocyte (x 10^9^/L)	10.19 ± 1.089	10.48 ± 1.033	9.81 ± 1.144	0.827
Neutrophils (x 10^9^/L)	4.588 ± 0.942	4.604 ± 0.915	4.573 ± 0.969	0.729
Lymphocyte (x 10^9^/L)	1.084 ± 0.295	1.002 ± 0.380	1.156 ± 0.209	0.804
Hemoglobin (g/L)	142.9 ± 1.481	136.5 ± 1.627	149.2 ± 1.334	0.681
CK (U/L)	4495.2 ± 586.34	11226 ± 1080	685.4 ± 92.68	<0.001
Myoglobin (ng/mL)	627.3 ± 71.40	852.1 ± 79.14	579.7 ± 63.66	0.010
Urine output (mL/d)	1215 ± 123.3	1200 ± 145.9	1224 ± 100.6	0.890
Creatinine (umol/L)	179.6 ± 24.02	176.4 ± 28.58	181.8 ± 19.46	0.873
Urea nitrogen (mmol/L)	11.50 ± 1.250	10.83 ± 1.343	11.92 ± 1.157	0.549
eGFR (Ml/min/L)	59.16 ± 7.876	58.76 ± 8.951	59.38 ± 6.801	0.956
PCT (ng/mL)	2.491 ± 0.800	2.853 ± 0.992	2.293 ± 0.599	0.610
CRP (mg/L)	42.11 ± 17.32	51.32 ± 20.87	35.32 ± 13.77	0.510

APACHEII Score: Acute Physiology and Chronic Health Evaluation II Score; CK: Creatine Kinase; eGFR: Glomerular Filtration Rate; PCT: Procalcitonin; CRP: C-Reaction Protein.

### Outcomes of subjects with RM-associated AKI after CRRT termination

After CRRT termination, we found that the length of CRRT was significantly longer in patients with CK < 5,000 U/L (7.830 ± 0.909 vs 4.879 ± 0.792, *p* = 0.027). Although in-hospital mortality was not significantly different between the two groups (27.27% vs 22.64%, *p* = 0.389). The in-hospital length of stay, as well as ICU length of stay, was significantly shorter in the experimental group (11.88 ± 1.469 vs 16.42 ± 1.290, *p* = 0.026; 7.545 ± 0.866 vs 10.11 ± 0.793, *p* = 0.038). In addition, the MODS incidence was also similar (51.52% vs 49.06%, *p* = 0.064), which suggests that higher CK levels would not predict the onset of MODS Table 4.

**Table 4.** Outcomes of subjects with RM-associated AKI after CRRT termination.

Outcomes	All (*n* = 86)	C*K* > 5000 U/L (*n* = 33)	C*K* < 5000 U/L (*n* = 53)	*P* value
Length of CRRT periods	6.698 ± 0.850	4.879 ± 0.792	7.830 ± 0.909	0.027
In-hospital mortality	21 (24.42)	9 (27.27)	12 (22.64)	0.389
In-hospital stay length	14.67 ± 1.380	11.88 ± 1.469	16.42 ± 1.290	0.026
ICU stay length	9.128 ± 0.830	7.545 ± 0.866	10.11 ± 0.793	0.038
MODS incidence	43 (50.00)	17 (51.52)	26 (49.06)	0.064

CK: Creatine Kinase; CRRT: Continuous Renal Replacement Therapy; ICU: Intensive Care Unit; MODS: Multiple Organ Dysfunction Syndrome.

### Outcomes of subgroups with CK > 5,000 U/L after CRRT termination

Out of the 33 patients with CK above 5,000 U/L after CRRT termination, there were 13 cases with CK > 15,000 U/L, with the remaining cases within 5,000–15,000 U/L. These participants presented a similar length of CRRT (*p* = 0.079), as well as in-hospital mortality (*p* = 0.579). In addition, the in-hospital and ICU length of stay were also comparable between these two subgroups (*p* = 0.086, *p* = 0.099). Finally, there was no higher incidence of MODS observed for patients with severe RM after CRRT termination (*p* = 0.090) Table 5.

**Table 5.** Outcomes of subgroups with CK > 5000 U/L after CRRT termination.

Outcomes	C*K* > 15000 U/L (*n* = 13)	CK: 5000–15000 U/L (*n* = 20)	*P* value
Length of CRRT periods	3.154 ± 0.741	6.000 ± 1.161	0.079
In-hospital mortality	5 (38.46)	4 (20.00)	0.579
In-hospital stay length	8.923 ± 1.542	12.80 ± 1.445	0.086
ICU stay length	5.769 ± 1.340	8.700 ± 1.081	0.099
MODS incidence	7 (53.85)	10 (50.00)	0.090

CK: Creatine Kinase; CRRT: Continuous Renal Replacement Therapy; ICU: Intensive Care Unit; MODS: Multiple Organ Dysfunction Syndrome.

### Correlations between CK and other variables after CRRT termination

**Figure 1.**
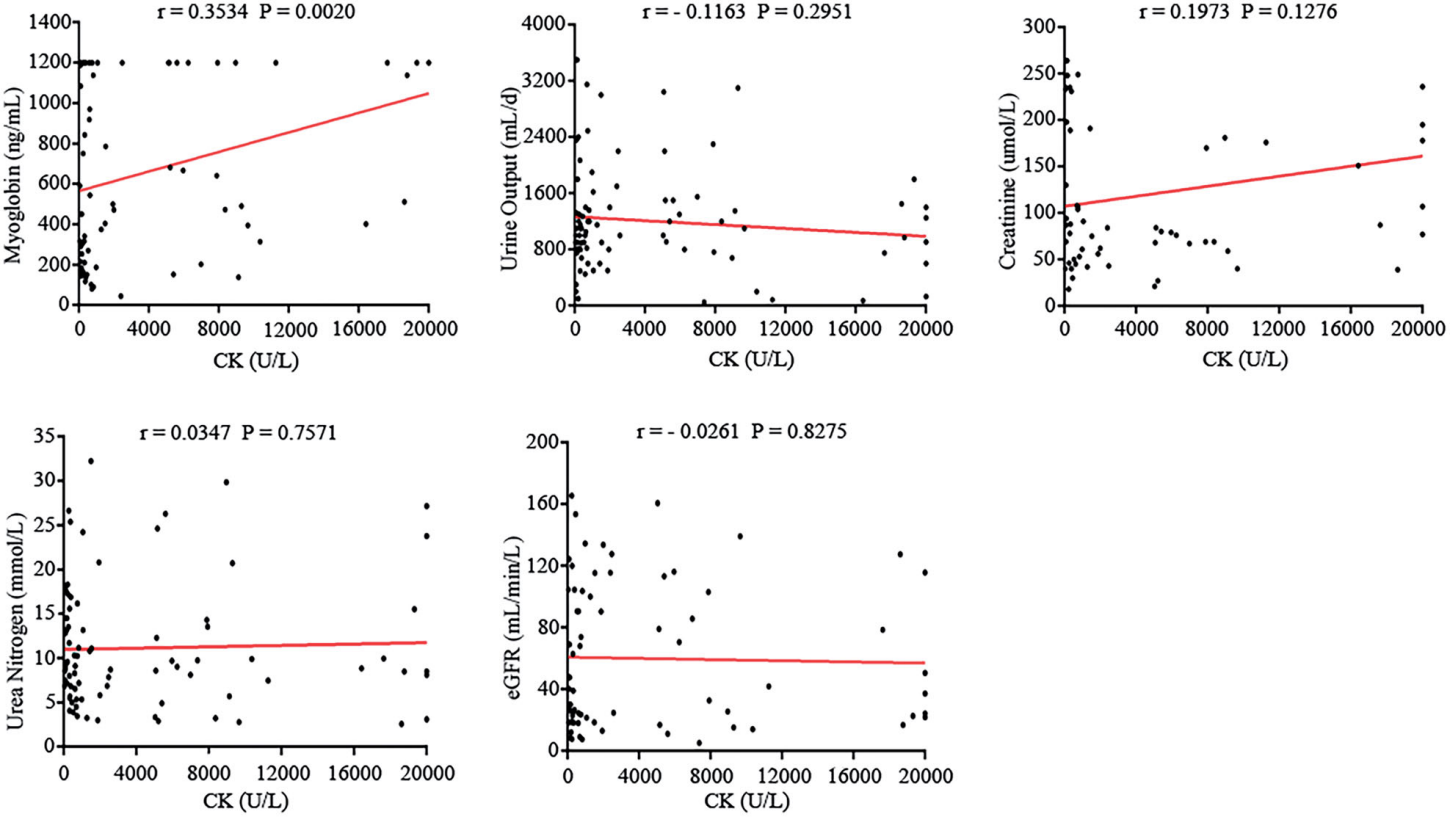
Correlation between CK and Myoglobin, Urine Output, Creatinine, Urea Nitrogen, and eGFR.

Since CK levels were significantly higher in the experimental group, we performed a linear correlation between CK and other variables after CRRT termination. The results of this study suggested a positive correlation between CK and myoglobin (*r* = 0.3534, *p* = 0.0020). For the renal function markers, such as urine output, creatinine, urea nitrogen, and eGFR, none of the above factors were associated with CK levels (all *p*-value > 0.05) (Figure 1).

## Discussion

RM is a syndrome characterized by rapid breakdown and leakage of skeletal muscle cell contents [19], such as myoglobin, CK, and other cytokines. During the past several decades, there have been multiple and diverse causes of RM, which include trauma, toxins, infections, drugs, and strenuous exercise [20–26]. All 86 cases in our study presented with certain classical symptoms, such as muscle weakness, myalgia, or dark-colored urine, along with significantly higher CK levels, and without red blood cells in the urine sediment. Our analysis showed that serum CK levels were not associated with an increased need for CRRT in patients with RM-associated AKI. In addition, there was a positive correlation between CK and myoglobin after CRRT termination.

Among various forms of RM, the most common pathophysiologic feature is attributed to a rise in intracellular ionized calcium from injured muscles, which brings a loss of the transcellular calcium gradient, and eventually leads to cell death [11]. The release of CK, myoglobin, and various electrolytes into the blood circulation is characteristic of a classic clinical presentation, as well as RM–induced AKI. RM itself is characterized by fluid sequestration, which decreases renal perfusion, activates the Renin-Angiotensin-Aldosterone System (RAAS), and leads to renal vasoconstriction [3]. In addition, RM-associated AKI is believed to be triggered by myoglobin, which is considered to be a toxin that causes renal dysfunction. Myoglobin potentially precipitates with Tamm-Horsfall protein, which may lead to the tubular obstruction. Additionally, the heme moiety released from myoglobin potentially promotes free radical formation, which accelerates direct and ischemic tubular injury. All of the above potentially contribute to RM–induced AKI [5].

According to the RIFLE classification system, all participants in our study belonged to the “Risk,” “Injury” or “Failure” stages of renal function. In order to prevent the deterioration of RM-associated AKI, traditional fluid resuscitation, including crystal and colloid liquid, was applied to all patients. For those with remarkable metabolic acidosis, sodium bicarbonate therapy was used to stabilize the homeostasis [27]. Additionally, CRRT was chosen because all patients with RM-associated AKI met at least one of the intervention criteria.

Data from several previous studies have supported the effectiveness of CRRT for RM- associated AKI. Participants in our study had a significantly high McMahon score, as well as worse renal function on admission, which suggested a need for renal replacement therapy. Despite the fact that CRRT is reputedly safe and efficacious for RM–induced AKI, its use is still controversial.

According to the British Renal Association guidelines for AKI, if the urine output is > 1,000 mL/D, and serum creatinine ≤ 265 μmol/L, termination of CRRT could be considered [18]. However, for RM-associated AKI, whether CK levels should be considered, when deciding whether CRRT termination is a suitable choice for a patient, has been uncertain. First, moderate RM with CK above 5,000 U/L predicts an increased risk of renal injury. Second, severe RM, which is characterized by CK > 15,000 U/L, shows a higher risk of dialysis. Lastly, patients with a higher level of CK predicts potential ongoing muscle injury or incomplete recovery after treatments.

Two retrospective reviews of 30 and 41 cases based on exertional RM-associated AKI, found discharged CK values ranging from 1,410 to 94,665 U/L and 10 to 61,617 U/L [30,31]. Mario Pezzi et al. reported a case analysis related to the use of coupled plasma filtration adsorption (CPFA) in traumatic RM; the lowest CK level was 9,000 U/L, and the highest level was 20,000 U/L when CPFA stopped [11]. Olcay Dilken et al. reported achievement of a successful reduction of CK levels to 40,000 U/L in severe RM using extracorporeal blood purification (CytoSorb) after termination [12]. Recently, an article published by Eka Laksmi Hidayati et al. reported a progressive reduction of CK levels to 61 U/L for RM caused by multiple wasp stings through continuous plasma exchange [29]. To our knowledge, there is still no consensus on confirmed CK levels that should be reduced by CRRT.

In our study, we investigated whether CK levels should be considered as an indicator for CRRT termination in patients with RM-associated AKI. Patients with different CK levels were included into two groups, and those with CK > 5,000 U/L after termination were included in the experimental group. The APACHEII and McMahon scores were not significantly different between the two groups on admission, which suggested a similar metabolic state among all participants. Laboratory examinations, such as routine blood tests, CK levels, myoglobin, renal function indicators, and inflammatory biomarkers, were comparable between the two groups on admission. After CRRT termination, the APACHEII score, as well as the McMahon score, also revealed no significant differences. The average McMahon score for all patients was below six, which predicted a lower requirement for renal replacement therapy further [3]. We also found that serum myoglobin was significantly higher in the experimental group, with high levels of CK. The renal function indicators, such as urine output, creatinine, urea nitrogen, and eGFR, along with inflammatory biomarkers, were consistent between after CRRT. Although in-hospital mortality showed no difference, the length of stay for in-hospital, as well as ICU, was significantly lower, and the CRRT period was significantly shorter for the higher CK level group. First, a limited level of CK could be reduced by each CRRT cycle, which is based on the lifetime of filters for hemodialysis, whole body inflammatory levels, and the speed of blood flow. Second, to further reduce the CK level to below 5,000 U/L, the length of CRRT cycles was significantly increased in the control group. Lastly, to guarantee the safety of CRRT during whole treatments, the in-hospital and ICU stay lengths were increased in turn.

It is interesting to note that CK levels after CRRT termination had no relationship to in-hospital mortality or MODS incidence. Two points may explain this result: First, patients included in this study were strictly screened, the urine output after CRRT termination was above 1,000 mL/D, and serum creatinine was below 265 μmol/L. Although the CK levels after CRRT termination might be associated with potential ongoing muscle injury or incomplete recovery, a relatively healthy renal function could guarantee the elimination of catabolic products, without CRRT continuation. Second, despite a higher CK level in the experimental group, comparable APACHEII and McMahon scores between the two groups after CRRT represented controlled homeostasis. As a result, there showed no significant difference between in-hospital mortality and incidence of MODS.

Another important finding was a positive correlation between CK and myoglobin after CRRT treatment. However, there was no evidence that CK had any relationship to renal function indicators. To the best of our knowledge, there is an accumulation of CK and myoglobin in the blood after muscle damage. CK reaches its maximum value after around 24 h, and is then eliminated by oxidation in the blood. This process is independent of liver and kidney function. Myoglobin reaches its maximum value within 12 h and is rapidly cleared by the kidneys. If kidney function is unrestricted [1], CK and myoglobin are sensitive indicators for striated muscle injury. For patients with RM-associated AKI, a large amount of myoglobin is released into the blood, which exceeds renal excretion capacities and causes nephrotoxic effects. In this case, the levels of CK and myoglobin may show a consistent exponential growth with each other, along with persistent muscle damage. CRRT, an approach to aggressively remove the serum uremia-related molecules (such as creatinine and urea nitrogen), mainly aims to protect renal function, while its effect on sustained muscle injury is limited [28]. So the results of our study suggest that there is no relationship between CK and renal function markers after CRRT by linear correlation analysis.

Lastly, we performed a subgroup analysis for patients with CK > 5,000 U/L, since it included those with severe RM at the end of CRRT. It is interesting to note that neither in-hospital mortality nor length of stay showed any difference among subgroups. These results suggest that if renal functions are significantly improved by CRRT, CK levels could be gradually returned to normal through traditional supportive treatments, such as fluid resuscitation and alkalization of urine. Importantly, on the premise of improved renal functions, a higher CK level might not be an independent risk factor for in-hospital mortality.

There were several potential limitations associated with the research presented here that need to be highlighted. First, our study only divided patients into two groups according to CK levels after CRRT termination. We could not identify an appropriate range that CK should be decreased to that would guarantee low recurrence of RM–induced AKI. Second, since there was a positive correlation between CK and myoglobin at the end of CRRT, it is unclear whether high levels of myoglobin would lead to a poor prognosis, and this requires further investigation. Lastly, as this was a single center and retrospective analysis with a limited number of cases, selection bias cannot be ruled out. In addition, with the development of new and more advanced therapeutics over the past decade, relative biases cannot be eliminated. It is possible for us to hold a larger, randomized and controlled trial over an extended period of time to carry on future research in this area.

## Conclusion

For patients with rhabdomyolysis-associated AKI, CRRT termination may be independent of CK levels, if renal functions have recovered to an appropriate level. Prospective clinical trials would be needed to investigate and confirm the optimal CK range that could be used as a guideline to help prevent the recurrence of renal impairments after treatments.

## Data Availability

The datasets used and/or analyzed during the current study are available from the corresponding author on reasonable request.
